# Supplementary material for: Content-rich biological network constructed by mining PubMed abstracts
Source: BMC Bioinformatics. 2004 Oct 8;5:147. doi: 10.1186/1471-2105-5-147 (PMC528731; doi:10.1186/1471-2105-5-147)
Supplement: Additional File 5 — The original Chilibot query results of the term "long-term potentiation (LTP)" and 22 other terms, limiting the latest references analyzed to the years 1990, 1995, 2000, and 2004. [file 1471-2105-5-147-S5.bz2 › chilibotAdditionalFile5/ltp2004/html/ERK_ACTIN.html]

 


 **ERK** and **ACTIN** 
  
Found 207 abstracts in PubMed,  **30 abstracts were retrieved and analyzed**.  


---

 Search Google  |
 PDF files only 
|  EDU domain only 

---

**Interactive relationship** (e.g. stimulation, inhibition, etc)

- Phosphorylated  **ERK**  and p38 MAPK were not colocalized with F  **actin**  in neutrophils stimulated by cytokines or FMLP.  Ref: 12954601 Am J Physiol Cell Physiol, 2004
- These results suggest that the activation of  **ERK** , JNK and p38 MAP kinases is involved in 4 HNE mediated  **actin**  remodeling, and endothelial barrier function.  Ref: 14699126 J Biol ChemJ Biol Chem, 2004
- The glutamate induced declustering of Cupidin preceded that of F  **actin**  and drebrin, was elicited by NMDA receptor mediated calcium influx, and was followed by a downstream pathway including MAPK  **ERK**  and protein tyrosine kinase.  Ref: 14511114 J Neurochem, 2003
- TNF and GM CSF induced  **actin**  depolymerization and morphological changes... were partially inhibited by either PD 98059, an inhibitor of mitogen activated protein kinase MAPK extracellular signal regulated kinase  **ERK**  kinase, or SB 203580, an inhibitor of p38 MAPK, and were almost completely abolished by these inhibitors in combination.  Ref: 12954601 Am J Physiol Cell Physiol, 2004
- 3 immunoblotting of proteins phosphorylated on SP K motif suggests the presence of  **ERK**  substrates in the brush border, one of which could be  **actin** .  Ref: 14575703 Biochem Biophys Res Commun, 2003

**Parallel relationship** (e.g. studied together, co-existance, homology, etc.)

- Moreover, ANXA1 reduces proliferation by  **ERK**  mediated disruption of the  **actin**  cytoskeleton and ablation of cyclin D1 protein expression and not by  **ERK**  mediated induction of the cyclin dependent kinase, CDK2, inhibitor p21 cip waf.  Ref: 14516791 Exp Cell Res, 2003
- These findings suggest that TNF, GM CSF, and G CSF induce  **actin**  depolymerization and morphological changes through activation of  **ERK**  and or p38 MAPK.  Ref: 12954601 Am J Physiol Cell Physiol, 2004
- The authors conclude that the early activation of RhoA is essential for stretch induced  **actin**  stress fiber formation and  **Erk**  activation in MC, events which are prevented by NO and cGMP through their action on RhoA.  Ref: 14569089 J Am Soc Nephrol, 2003
- We previously reported that the Rho Rho kinase pathway controls cyclin D1 expression by preventing its early G1 phase induction in response to Rac and or Cdc42, thus increasing its dependence on  **ERK**  signaling and  **actin**  stress fiber formation.  Ref: 12919678 Dev Cell, 2003
- The Rho kinase inhibitor Y 27632 attenuated  **Erk**  activation in a dose dependent manner and prevented stretch induced  **actin**  stress fiber formation.  Ref: 14569089 J Am Soc Nephrol, 2003
- Since  **ERK**  activation appears to be independent of the integrity of  **actin**  filaments, as cytochalasin D and latrunculin A did not block  **ERK**  activation, it can be concluded that LPS endocytosis is independent of  **actin**  cytoskeleton remodeling as is the case in mammalian systems.  Ref: 14563359 Insect Biochem Mol Biol, 2003
- NO and cyclic GMP abrogate stretch induced  **Erk**  activation by inducing  **actin**  cytoskeletal disassembly.  Ref: 14569089 J Am Soc Nephrol, 2003
